# Effect of yoga on mental health: Comparative study between young and senior subjects in Japan

**DOI:** 10.4103/0973-6131.78173

**Published:** 2011

**Authors:** Derebail Gururaja, Kaori Harano, Ikenaga Toyotake, Haruo Kobayashi

**Affiliations:** Department of Medical Welfare, Kawasaki University of Medical Welfare, Okayama, Japan; 1 Department of Welfare System and Health Science, Okayama Prefectural University, Okayama, Japan; 2 Department of Welfare System and Health Science, Yufukai Medical Corporation, Okayama, Japan

**Keywords:** Salivary amylase activity, state trait anxiety inventory, stress, yoga

## Abstract

**Background:**

Japan has a large number of senior citizens. Yoga can be wisely applied in old age care. There is no any age restriction to practice yoga. The effect may differ by age. There is a need to study the mechanism of action of yoga with respect to age.

**Aim:**

This study was conducted in Japan to find the effect of yoga on mental health between young and senior people.

**Materials and Methods:**

Twenty-five normal healthy volunteers of both sexes were divided into two groups according to age. Fifteen participants of the age group between 65 to 75 years and 10 participants of the age group between 20 to 30 years were selected. This study was approved by the ethical committee of Kawasaki University of Medical Welfare. Selected individuals were subjected to 90 min of yoga classes once or twice a week for a month. Salivary amylase activity was assessed before and after yoga practice. State Trait Anxiety Inventory (STAI) was given before yoga on the first day and after one month of practice to assess the change in State anxiety and Trait anxiety.

**Results:**

Senior group – Salivary amylase activity decreased from 111.2±42.7 to 83.48±39.5 kU/L [average±standard deviation]. Younger group – Salivary amylase activity reduced from 60.74±31.8 to 42.39±24 kU/L. Senior group – State anxiety score decreased from 41.13 ±8.43 to 30.8±6.49, Trait anxiety score reduced from 45.66±7.5 to 40.73±8.3. Younger group – State anxiety score reduced from 38.7±4.8 to 30.8±4.1,Trait anxiety score reduced from 46.2±7.9 to 42.9±9.1. Changes were statistically significant with *P*<0.05.

**Conclusion:**

Decrease in Salivary amylase activity may be due to reduction in sympathetic response. Reduction in State and Trait anxiety score signifies that yoga has both immediate as well as long-term effect on anxiety reduction. Thus yoga helps to improve the mental health in both the groups.

## INTRODUCTION

Japan is a country with a large number of senior citizens. In the year 2000, the population of people aged above 65 years was 17.3% and in 2009 it has reached 22%.[1] Cancer and heart diseases are the two major causes of death in Japan. Dementia, stress, degenerative joint diseases and respiratory infections are the most common health problems affecting the elderly population. Daycare centers and old age homes are working hard to improve the quality of life of the aged. Different methods are being adopted to improve their lifestyle and keep them active. Music therapy, animal therapy, massage and stretch exercises are among them.

Yoga is an ancient Indian science which helps to improve physical, mental, social and spiritual health. Yoga has found its special existence in Japan by its peculiarities like asana and pranayama. But many consider yoga as an alternate to exercise. Few research works have been conducted in Japan on yoga. There is a need to show that yoga is not merely an exercise system but it has many more health benefits. It can be wisely applied in the old age care to improve the quality of life. Research is needed to understand the mechanism of action of yoga.

Stress is a major factor affecting the mental health of a person irrespective of age. Presentation of the stress may vary from that of fight to flight phenomenon. Chronic stress is the major cause of many physical and mental disorders.[2] Yoga has been effectively used in the management of stress. It has been observed that the practice of yoga decreases verbal aggressiveness compared to physical exercise.[3] It is also useful against physical stress like cold exposure[4] and stress due to diseases like epilepsy.[5] Yoga has been found useful for mental disorders like depression.[6] In a study conducted by Oken *et al*., on healthy seniors participants were divided into three groups as yoga, exercise and waitlist control. The yoga group showed significant improvement in quality of life and physical measures compared to the exercise and waitlist control group.[7]

Age is an important factor to be considered while studying the physiological changes. Even though there is no age restriction to practice yoga the effect may differ by age. There is a need to study the effect of yoga with respect to age.

Assessment of salivary alpha amylase (sAA) activity is a newly developing, noninvasive, simple method to assess the acute sympathetic response or stress.[8] No study has been conducted in Japan on the effect of yoga on sAA. The aim of this study is to compare the effect of yoga on mental health between young and senior subjects in Japan.

## MATERIALS AND METHODS

### Subjects

In this study 25 normal healthy volunteers of both sexes, interested in yoga were selected. They were divided into two groups according to age. First group with 15 participants of age group between 65 to 75 years and a second group of 10 individuals between the age group of 20 to 30 years were selected.

#### Inclusion criteria

1. No previous experience in Yoga
2. In senior group - Controlled hypertension

#### Exclusion criteria

1. Severe systemic illnesses like severe hypertension and insulin-dependent diabetes mellitus
2. Any painful condition like arthritis
3. Recently undergone major surgery

#### Ethical clearance

Informed written consent was taken from all the participants.

This study was approved by the ethical committee of Kawasaki University of Medical Welfare, Kurashiki, Okayama prefecture, Japan.

#### Design of the study

It is a comparative study between the young and senior subjects to assess the effect of yoga on mental health. Fifteen seniors and 10 young participants were assessed before and after yoga practice.

### Methods

Selected individuals were subjected to 90 min of yoga classes once or twice a week for a month. Yoga classes were conducted in the morning between 10 am to 12 noon, minimum of one hour after taking food. They were instructed to practice Asanas, Pranayama and Meditation. Yoga program was designed based on:

1. Postures should be simple and safe
2. Should give stretch to the muscles of the extremities, trunk and neck
3. Should be performed in all postures: standing, sitting, supine and prone. Asanas were -

#### Standing position

1. Tadasana,
2. Ardha katichakrasana,
3. Pada hasthasana,
4. Trikonasana.

#### Sitting position

1. Vakrasana,
2. Vajrasana,
3. Paschimottanasana,
4. Gomukhasana.

#### Supine position

1. Pavanamuktasana,
2. Pada uttanasana - Eka and Dwipada.

#### Prone position

1. Bhujangasana,
2. Shalabhasana - Eka and Dwipada.

At the end of asana session, they were advised to practice Shavasana for 5 min.

After asanas, pranayama was practiced

1. Kapalabhati - 3 rounds each - 30 to 50 strokes,
2. Nadishodana pranayama - 3 rounds,
3. Bhramari -3 rounds, followed by ‘OM’ meditation for 15 min.

### Assessment criteria

1. Salivary amylase activity was assessed before and after yoga practice once or twice a week for a month. Salivary amylase activity monitor or Cocorometer of Nipro Corporation, Osaka, Japan was used to measure salivary amylase activity. The measurement system consists of a disposable test strip and the main unit of the measuring instrument. The test strip consists of the collection sheet in which the saliva collection paper is installed and the test paper holder. The test paper is affixed to the back side of the test paper holder. The saliva transcript mechanism and the optical measuring unit to measure the color density of the reagent are installed in the main section of the measuring instrument.

The collection sheet is inserted into the mouth and about 20 to 30 μl of the saliva in the sublingual region is directly collected for 10 to 30 sec, thereafter the test strip is placed in the main unit of the measuring instrument and the cover is closed. When the transcript lever is operated the test paper put on the reverse side of the test paper holder is pressed against the saliva collection paper and the saliva is transcribed. This time point is detected as the reaction start time and the alarm sounds when the set transcript time has passed. The collection sheet is then removed by lifting the lever. The color density of the test paper due to the enzyme reaction is measured using the optical device. The color density after the previously set time from the reaction starting is measured. The amylase activity value converted from the measured value is indicated on the display.[9]

2. State Trait Anxiety Inventory (STAI) was given before yoga practice and after one month of yoga practice to assess change in State anxiety and Trait anxiety. The Japanese version of Form X was used, X-1 form for State anxiety that evaluates how respondents feel right now. Trait anxiety form X-2, assesses how people generally feel. Personality states are often transitory; they can recur when evoked by appropriate stimuli. In contrast personality traits can be conceptualized as relatively enduring differences among people in specifiable tendencies to perceive the world in a certain way or behave in a specified manner with predictable regularity. Each form contains 20 questions and each question is rated 1 to 4. In responding to the STAI S-Anxiety scale, examinees blacken the number on the standard test form to the right of each item statement, that best describes the intensity of their feeling 1) not at all, 2) somewhat, 3) moderately so, 4) very much so. In responding to the T-anxiety scale examinees were instructed to indicate how they generally feel by rating the frequency of their feeling of anxiety on the following four-point scale: 1) almost never, 2) sometimes, 3) often, 4) almost always. The questionnaire contains both anxiety questions (e.g. I feel frightened, I feel upset) and anxiety absent questions (e.g. I feel calm, I feel relaxed). The scoring weights for the anxiety present items are the same as the blackened numbers on the test form i.e. 1, 2, 3 and 4. The scoring for the anxiety absent items are reversed i.e. responses marked 1,2,3,4 are scored 4,3,2,1 respectively. Scores for both the S- and T-anxiety scales can vary from a minimum of 20 to a maximum of 80.[10]

### Statistical analysis

Obtained data was analyzed statistically using paired *t* test to test the significance within the group, and compared between the groups using unpaired t test to assess the difference between the groups. SPSS program and Microsoft Excel 2003 were used to perform statistical analysis. Level of significance was set at *P*<0.05.

## RESULTS

In senior group, out of 15 participants, 11 were females and four were males. In the 10 young participants, seven were females and three were males. In the senior group, five members were taking medicine for hypertension and it was under control.

Salivary amylase activity in the senior group – It decreased from111.2±42.7 to 83.48±39.5 kU/L [average±standard deviation]. The change which occurred after yoga practice was statistically significant.

Salivary amylase activity in the younger group - It also reduced after yoga practice from 60.74±31.8 to 42.39±24 kU/L with *P*<0.05, a statistically significant change.

**Figure 1:**
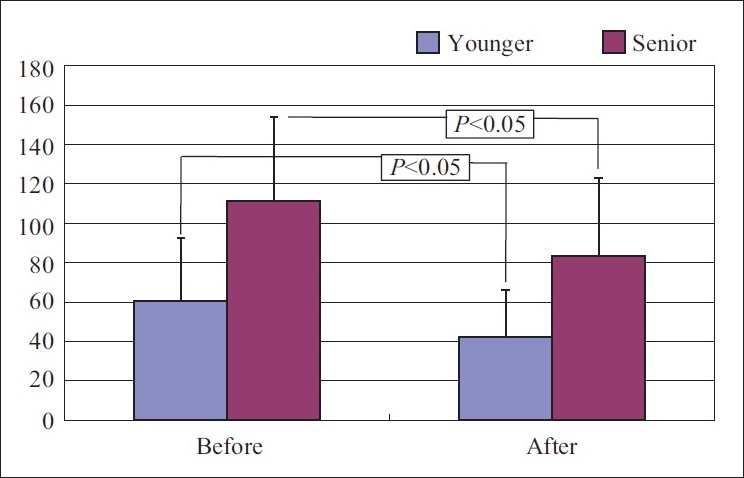
Salivary amylase activity before and after yoga practice in young and senior groups

**Figure 2:**
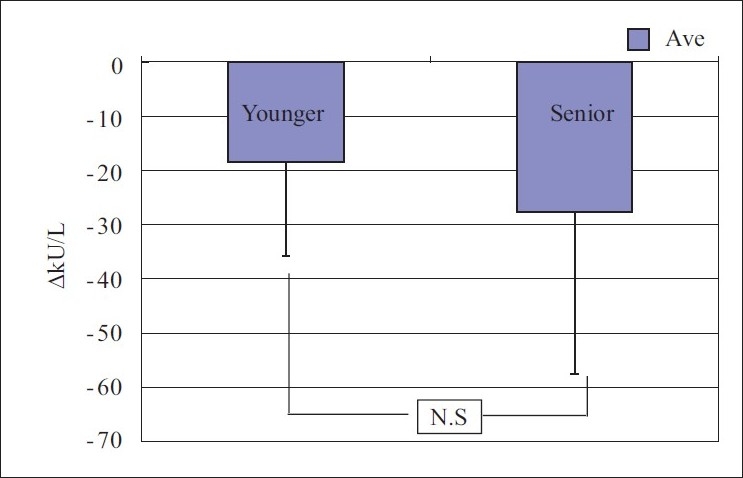
Change in salivary amylase activity as comparison between young and senior groups

When both the groups were compared to assess any difference in effect between the groups, it showed insignificant difference with *P*<0.05 [Figures 1 and 2].

### STAI - Senior group

#### State anxiety

Score decreased from 41.13±8.43 to 30.8±6.49, *P*<0.05 a statistically significant change.

#### Trait anxiety

Score for general feelings reduced from 45.66±7.5 to 40.73±8.3 with *P*<0.05, a statistically significant decrease.

When the change in state anxiety was compared with that of trait anxiety the difference was significant. The response was more for state anxiety.

### STAI - Younger group

#### State anxiety

Score decreased from 38.7±4.8 to 30.8±4.1. The change which occurred after yoga practice in present feeling was statistically significant.

#### Trait anxiety

**Figure 3:**
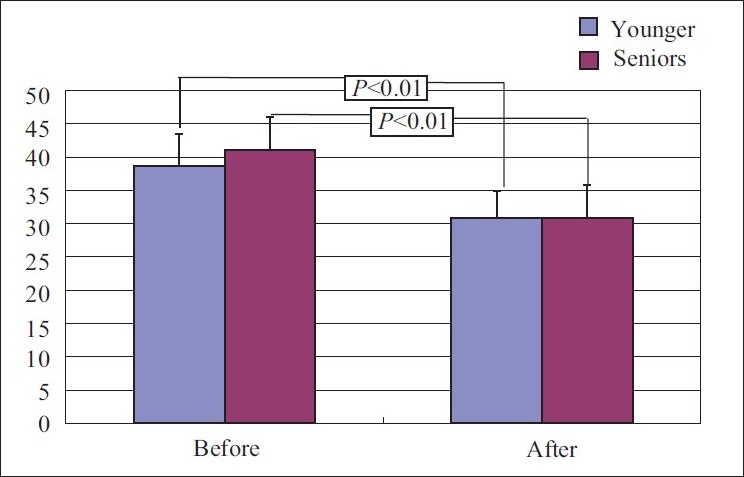
State anxiety score before and after yoga in young and senior groups

**Figure 4:**
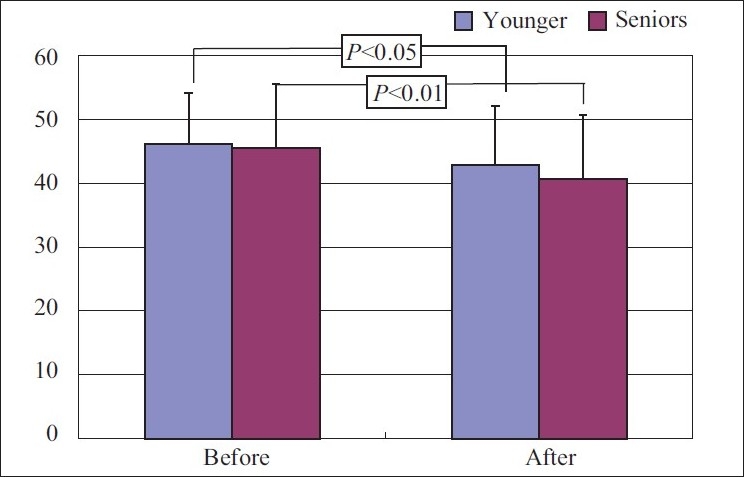
Trait anxiety score before and after yoga in young and senior groups

The score reduced from 46.2±7.9 to 42.9±9.1 with *P*<0.05, a significant change was observed [Figures 3 and 4].

When the change in state anxiety was compared with that of trait anxiety the difference was significant. Response was more for state anxiety compared to trait anxiety.

**Figure 5:**
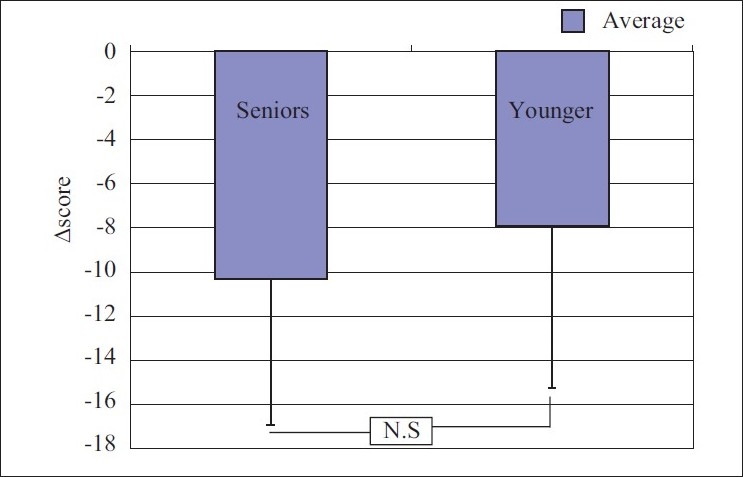
Comparison between the groups: Change in state anxiety score

**Figure 6:**
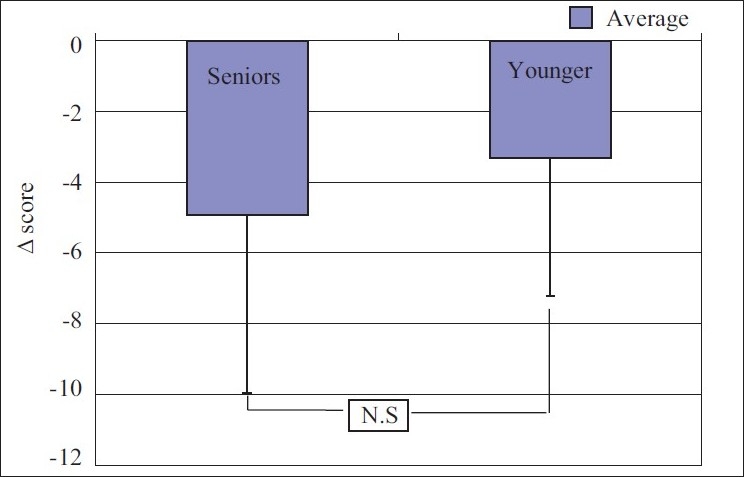
Comparison between the groups: Change in trait anxiety score

When compared between the groups for any difference in response for state anxiety and trait anxiety, the result was not significant [Figures 5 and 6].

## DISCUSSION

Salivary amylase activity is one of the simplest methods to assess the sympathetic activity. Alpha amylase is one of the major protein components of saliva. Sympathetic stimulation increases the salivary protein secretion and parasympathetic stimulation increases the salivary flow rate. It has been studied that stress-induced increase in salivary amylase is independent of salivary flow rate.[11] Decrease in sAA is due to reduced sympathetic stimulation. Reports are available about physical (exercise) and psychological stress increasing the alpha amylase level. It has been observed that stress induced increase of sAA and nor epinephrine levels are positively associated.[8] Normal value for sAAis not yet determined. Many factors will influence its activity[12] such as effect of time of day; values are lowest in the morning and reach highest levels in the evening. Gender – salivary amylase levels have not been shown to differ according to gender. Food – stimulation from smelling, tasting and ingesting food increases sAA. Age–Basal levels appear to remain stable over adult years, but it is not clear how they change in old age. Considering these points in this study, the time of yoga practice was fixed in the morning, about 90 min after taking food. As it is a comparative study the subjects were divided into two groups according to age to minimize the influence of the age factor.

In this study sAA level decreased after yoga practice in both the groups. When compared between the groups there was no difference in effect. In seniors sAA level was higher; this may be due to stress or increased sympathetic activity or increased epinephrine levels compared to the young. In two senior subjects in the initial two to three classes the sAA increased after yoga, this might be due to pain after practicing asana or failure to relax during meditation or anxiety about yoga. However, after a few classes, once they got accustomed to yoga, sAA levels came down. Decreased sympathetic activity signifies a decrease in stress level. In young individuals the sAA level was low compared to seniors and it reduced after yoga practice. This signifies that yoga helps to improve mental health and to overcome routine stress.

It is well documented that sympathetic adrenal activity increases during submaximal incremental exercise, especially once the anaerobic threshold has been reached.[13] Studies have shown that the anaerobic threshold can be detected through analysis of exercise-induced changes in sAA levels.[14] Changes in salivary amylase, blood lactate level, electromyographic response of working muscles and relation of sympathetic response to exercise was also studied.[15] Previous studies have also shown that yoga-based guided relaxation reduces sympathetic activity.[16] Even mentally repeating ‘OM’ showed reduction in sympathetic activity compared to a neutral word and non-targeted thinking.[17] In this study subjects practiced relaxing asanas, pranayama and meditation. Reduction in sAA may be due to decreased sympathetic response. As sAA is an indicator of acute changes in the sympathetic activity this result show that yoga immediately reduces the sympathetic response or stress and relaxes the body and mind.

STAI was used as subjective criteria to assess the effect of yoga on mental health. Trait anxiety refers to relatively stable individual differences in anxiety-proneness, that is, differences between people in the tendency to perceive stressful situations as dangerous or threatening and to respond to such situations with elevation in the intensity of their state anxiety reactions. T-anxiety may also reflect individual differences in the frequency and intensity with which anxiety states have been manifested in the past, and in the probability that S-anxiety will be experienced in the future. The stronger the anxiety trait, the more probable that the individual will experience more intense elevations in S-anxiety in a threatening situation. Scores on the S-anxiety scale increase in response to physical danger and psychological stress.[18]

Both state anxiety and trait anxiety scores decreased after yoga practice in both the groups. There was no difference in response between the groups. Both the young and seniors showed a decrease in their anxiety scores. Participants felt better and relaxed after practicing yoga. Response was more for state anxiety compared to trait anxiety. Thus yoga has both an immediate as well as long-term effect on anxiety reduction and helps to bring even behavior changes or controlled response to any type of stress if practiced regularly. It has been observed that yoga-based relaxation technique decreases state anxiety more in comparison to supine rest.[19] In one more study where yoga was a complementary treatment for depression, state anxiety score was decreased.[20]

Mechanism of action according to Yoga: Asana gives controlled stretch to the muscles and improves the flexibility. Relaxing asanas like shavasana help to relax the body and mind. Pranayama helps to gain control over the breathing. According to yoga, by controlling the prana one can control the mind.[21] By practicing asana, flow of prana becomes normal and by practicing pranayama one can control the prana. Even pranayama like Bhramari has a soothing effect on the mind. Later, by practicing meditation one can easily concentrate and relax. Chanting ‘OM’ helps to control the mind from different unwanted thoughts.

Lack of a control group may be considered as a limitation of this study but sufficient references about similar studies having control are provided so that the result of this study can be attributed to yoga.

## CONCLUSION

Yoga helps to improve the mental health of both the young and seniors by reducing stress. Yoga can be wisely applied in welfare programs to improve the Quality of Life in all age groups.
